# Large Chondrosarcoma of the Lower Rib Presenting as a Cystic Abdominal Mass

**DOI:** 10.5334/jbsr.3464

**Published:** 2024-03-27

**Authors:** Catherine Eeckhout Milants, Thomas Douchy, Mathieu Lefere

**Affiliations:** 1 Department of Radiology, Imeldaziekenhuis, Bonheiden, Belgium; 2 Department of Surgical Oncology, University Hospitals Leuven, Leuven, Belgium; 3 Radiology Department, Imeldaziekenhuis, Bonheiden, Belgium

**Keywords:** musculoskeletal, chondrosarcoma, rib, mimick

## Abstract

*Teaching point:* Chondrosarcoma of the lower rib may present with only minimal calcified chondroid matrix and may be misinterpreted as a liver lesion.

## Case History

A 65-year-old woman was referred for an ultrasound (US) exam of the liver because of elevated liver enzymes. Besides a feeling of abdominal pressure when sitting, there were no major clinical symptoms. The US showed a large right upper quadrant mass with a cystic appearance and an irregularly thickened wall (not shown). Because a large liver tumor was suspected, computed tomography (CT) was performed.

**Figure 1:**
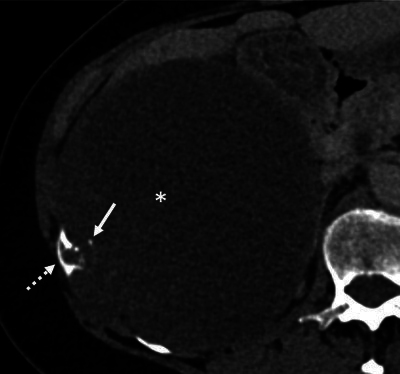
CT image showing the mass in the right hypochondrium.

**Figure 2:**
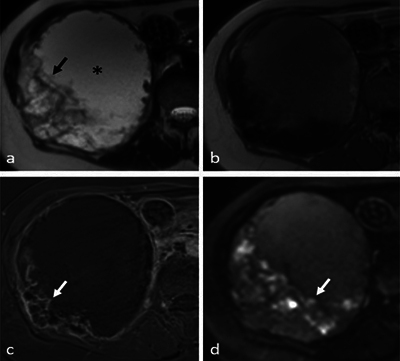
MRI images. **(a)** T2 weighted image. **(b)** T1 weighted image. **(c)** T1 weighted image with fat saturation. **(d)** Diffusion weighted image.

On CT, a large hypodense mass in the right hypochondrium was seen (Figure 1, asterisk). Remarkably, the anterior part of the 11th rib showed an irregular cortical defect in connection with the periphery of the lesion (Figure 1, dashed arrow), with small adjacent coarse calcifications (Figure 1, arrow). The right liver lobe and kidney were compressed without clear signs of organ invasion. On magnetic resonance imaging (MRI), the center of the mass appeared hyperintense on T2 (Figure 2a, asterisk) and slightly hyperintense on T1 (Figure 2b), suggesting a cystic nature. Extensive T2 hypo-intense wall thickening was also noted (Figure 2a, arrow), with peripheral contrast enhancement on T1 with fat saturation (Figure 2c, arrow). There was a diffusion restriction not corresponding to the enhancing solid components, most likely due to hemorrhagic content (Figure 2d, arrow). Mainly based on the CT findings, the diagnosis of chondrosarcoma of the right 11th rib was suggested. The patient was referred to a sarcoma referral center for treatment. A complete resection of the lesion was performed, with a partial resection of the right 10th, 11th, and 12th ribs and a partial resection of the right diaphragm. During surgery, the predominantly cystic nature of the mass was confirmed. Histopathological analysis revealed a primary chondrosarcoma with invasion of the 11th rib. It was graded as a grade 1 chondrosarcoma, although there was multifocal increased cellularity, possibly indicating an evolution to grade 2.

## Comments

Chondrosarcoma is the third most common primary malignant bone neoplasm, accounting for 20% to 27% of cases [1]. Primary chondrosarcomas are pathologically classified as low (grade 1) or high grade (grades 2–3), with treatment and prognosis depending on their grade. About 69% of primary chondrosarcomas in the axial skeleton are high grade. Although radiological grading of chondrosarcomas is challenging, there are some features suggesting a higher-grade lesion: moth-eaten or permeative bone destruction, or a large soft-tissue mass with little matrix mineralization. Chondrosarcoma of the lower rib can grow to a large size without symptoms and occasionally be difficult to differentiate from a peripheral liver mass on imaging. As shown in our case, osseous destruction and chondroid matrix calcification can be very subtle, relative to the size of the lesion.
